# High phosphate induces skeletal muscle atrophy and suppresses myogenic differentiation by increasing oxidative stress and activating Nrf2 signaling

**DOI:** 10.18632/aging.103896

**Published:** 2020-11-02

**Authors:** Lin-Huei Chung, Shu-Ting Liu, Shih-Ming Huang, Donald M. Salter, Herng-Sheng Lee, Yu-Juei Hsu

**Affiliations:** 1 Graduate Institute of Medical Sciences, National Defense Medical Center, Taipei, Taiwan; 2 Division of Nephrology, Department of Internal Medicine, Yuan Rung Hospital, Changhua, Taiwan; 3 Department of Biochemistry, National Defense Medical Center, Taipei, Taiwan; 4 Centre for Genomic and Experimental Medicine, Institute of Genetics and Molecular Medicine, University of Edinburgh, Edinburgh, UK; 5 Department of Pathology and Laboratory Medicine, Kaohsiung Veterans General Hospital, Kaohsiung, Taiwan; 6 Division of Nephrology, Department of Internal Medicine, Tri-Service General Hospital, National Defense Medical Center, Taipei, Taiwan

**Keywords:** hyperphosphatemia, muscle wasting, myogenic differentiation, oxidative stress, Nrf2

## Abstract

Skeletal muscle wasting represents both a common phenotype of aging and a feature of pathological conditions such as chronic kidney disease (CKD). Although both clinical data and genetic experiments in mice suggest that hyperphosphatemia accelerates muscle wasting, the underlying mechanism remains unclear. Here, we showed that inorganic phosphate (Pi) dose-dependently decreases myotube size, fusion index, and myogenin expression in mouse C2C12 skeletal muscle cells. These changes were accompanied by increases in reactive oxygen species (ROS) production and Nrf2 and p62 expression, and reductions in mitochondrial membrane potential (MMP) and Keap1 expression. Inhibition of Pi entry, cytosolic ROS production, or Nrf2 activation reversed the effects of high Pi on Nrf2, p62, and myogenin expression. Overexpression of Nrf2 respectively increased and decreased the promoter activity of *p62*-Luc and *myogenin*-Luc reporters. Analysis of nuclear extracts from gastrocnemius muscles from mice fed a high-Pi (2% Pi) diet showed increased Nrf2 phosphorylation in sham-operated and 5/6 nephrectomized (CKD) mice, and both increased p62 phosphorylation and decreased myogenin expression in CKD mice. These data suggest that high Pi suppresses myogenic differentiation *in vitro* and promotes muscle atrophy *in vivo* through oxidative stress-mediated protein degradation and both canonical (ROS-mediated) and non-canonical (p62-mediated) activation of Nrf2 signaling.

## INTRODUCTION

Muscle wasting, defined as loss of muscular mass and contractile capacity, is both a physiological consequence of normal aging and a common premature aging phenotype in patients with chronic kidney disease (CKD) [1]. Previous observational studies showed an association between muscle wasting and mortality in both pre-dialysis CKD patients and end-stage renal disease patients on dialysis [2, 3]. Because effective therapies to slow muscle wasting in such cases are lacking, further knowledge of the mechanisms involved in muscle wasting are required.

The kidney plays a critical role in regulating serum phosphate (Pi) levels. In the setting of kidney dysfunction, hyperphosphatemia may occur as a result of reduced Pi excretion due to declining glomerular filtration rate (GFR) [4]. Evidence suggesting that Pi is indeed a pro-aging factor received strong support from recent aging research using genetically engineered mouse models, which revealed a potential link between excess Pi and muscle wasting [5]. In mice, for example, both fibroblast growth factor 23 (FGF23) and the renal transmembrane protein Klotho act as Pi-regulating hormones whose genetic ablation leads to hyperphosphatemia and premature aging phenotypes, including muscle atrophy. Notably, muscle atrophy and other premature aging phenotypes were reversed in both Klotho- and FGF23-knockout mice by either a low-Pi diet or by genetic ablation of renal sodium-dependent Pi transporter solute carrier family 34, member 1 (NaPi2a) [5–7]. The molecular mechanisms underlying high Pi-induced muscle atrophy are not yet fully understood, but there is increasing evidence that oxidative stress and activation of the Nrf2/Keap1/p62 pathway may be involved.

Increased oxidative stress characterizes a variety of disorders, including cancer, heart failure, chronic obstructive pulmonary disease, and kidney disease, all of which are associated with skeletal muscle atrophy. Excess production of reactive oxygen species (ROS) may inhibit muscle protein synthesis and promote protein breakdown, leading to muscle atrophy [8]. Accumulation of ROS under oxidative stress conditions is linked to canonical activation of nuclear factor erythroid 2-related factor 2 (Nrf2), a sensor of oxidative stress [9], by preventing binding to its native repressor Kelch-like ECH-associated protein 1 (Keap1). Keap1 is an adapter substrate of Cullin 3 (Cul3) ubiquitin E3 ligase complex and represses Nrf2 activity through sequestration, ubiquitination, and proteasomal degradation under basal conditions. During oxidative stress, elevated ROS generation triggers oxidation of specific cysteine residues in Keap1. This reduces Nrf2 ubiquitination and promotes its nuclear translocation and activation [10]. Moreover, recent research demonstrated that oxidative stress induces *Nrf2* gene expression and transcriptional activity in C2C12 skeletal muscle cells and in rodent skeletal muscles [11–13]. In turn, inhibition of Nrf2 using small interfering RNA (siRNA) enhanced, while upregulation of Nrf2 using sulforaphane inhibited, myogenic differentiation of C2C12 cells [12, 13]. Notably, high Pi concentrations *in vitro* reportedly induce oxidative stress by altering mitochondrial membrane potential (MMP) and oxidative phosphorylation in insulin-secreting cells [14, 15] and endothelial cells [16]. These data suggest that oxidative stress-mediated Nrf2 activation may play a role in the pathogenesis of muscle wasting induced by high Pi.

p62 is a stress-inducible, multifunctional protein. Accumulation of p62-enriched, polyubiquitinated misfolded proteins leads to inclusion body formation. In addition, p62 can also target ubiquitinated proteins to the autophagic machinery for lysosomal degradation [17]. Recent studies have shown that age-related skeletal muscle atrophy is characterized by abnormal p62 accumulation [18]. In mice, muscle-specific deletion of the autophagy-related genes Atg5 or Atg7 results in skeletal muscle atrophy accompanied by accumulation of p62 [19, 20]. Furthermore, various stress conditions induce phosphorylation of p62 at a serine residue within its Keap1-interacting region (KIR), which markedly enhances its binding affinity for Keap1. As a result, phosphorylated p62 competitively inhibits the interaction between Nrf2 and Keap1, leading to non-canonical activation of Nrf2 [10]. This evidence suggests a possible link between p62 and Nrf2/Keap1 in the pathogenesis of muscle wasting.

The aim of the present study was to determine the influence of hyperphosphatemia on both skeletal muscle cell differentiation *in vitro* and on CKD-associated muscle wasting *in vivo*. We hypothesized that hyperphosphatemia induces muscle wasting by increasing oxidative stress, p62 phosphorylation, and activation of Nrf2 transcriptional activity. To test this hypothesis, we determined the effects of high Pi on myogenic differentiation, generation of mitochondrial and cytosolic ROS, and expression of Nrf2, Keap1, p62 and the myogenic differentiation marker myogenin in C2C12 skeletal muscle cells. To evaluate the effect of high Pi *in vivo*, we measured serum markers of Pi homeostasis, grip strength, and Nrf2, p62, and myogenin expression in gastrocnemius (GA) muscle samples from sham-operated and CKD mice fed a normal Pi or a high Pi diet. Our findings implicate both canonical and non-canonical activation of Nrf2 as prominent mechanisms contributing to CKD-associated muscle atrophy.

## RESULTS

### High Pi stimulation suppresses differentiation of C2C12 skeletal muscle cells

**Figure 1:**
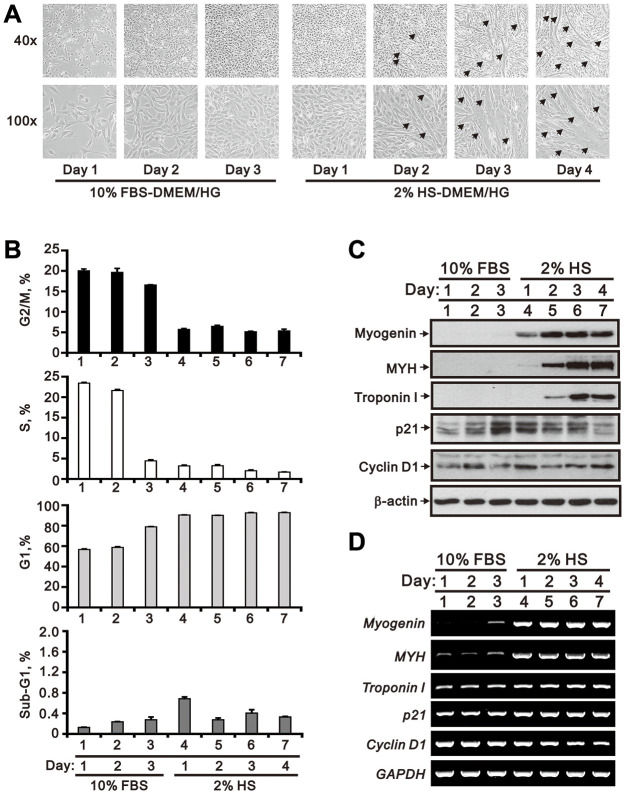
**Morphological characteristics of proliferating and differentiating C2C12 cells *in vitro*.** Mouse C2C12 myoblasts were cultured for 3 days in growth medium (DMEM/HG plus10% FBS), followed by 4-day incubation in differentiation medium (DMEM/HG plus 2% HS). (**A**) Morphological changes in C2C12 cells during the time course of proliferation and differentiation. Representative micrographs were obtained using a light microscope at 40x (upper row) or 100x (lower row) magnification. The arrows indicate mature, multinucleated myotubes. (**B**) Cell cycle phase distributions during the course of C2C12 growth and myogenesis. (**C**) Whole-cell lysate immunoblots from cultured C2C12 cells assessing the expression of myogenic differentiation markers (myogenin, MYH, and troponin I) and cell cycle regulators (p21 and cyclin D1). β-actin was used as loading control. (**D**) RT-PCR analysis of *myogenin*, *MYH*, *troponin I*, *p21* and *cyclin D1* mRNA in cultured C2C12 cells. *GAPDH* was used as loading control. Data are presented as means ± SEM.

Under normal differentiating conditions (2% HS), a time-dependent increase in the number of multinucleate myotubes was observed in cultured C2C12 cells (Figure 1A). During this period, cell cycle phase distribution analysis indicated a predominant G1 phase arrest and decreased S and G2/M phases (Figure 1B). In parallel, upregulation of differentiation markers, including myogenin, myosin heavy chain (MYH), and troponin I, was observed at both the mRNA and protein levels (Figure 1C and 1D). Meanwhile, although protein levels of p21 and cyclin D1 varied, levels of p21 mRNA were increased, while levels of cyclin D1 mRNA were decreased in differentiating C2C12 cells (Figure 1C and 1D).

**Figure 2:**
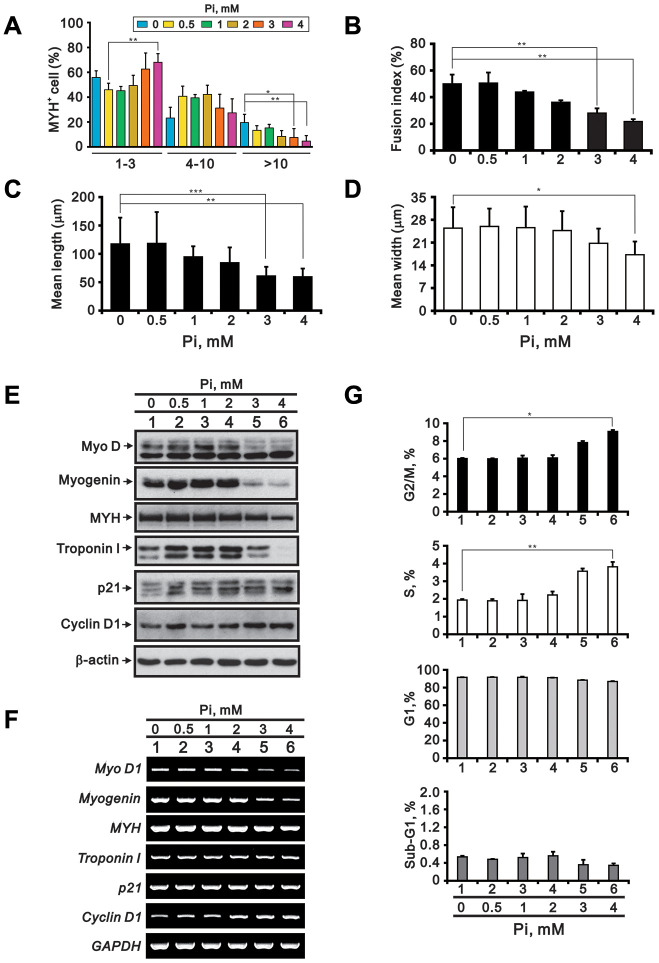
**High Pi impairs C2C12 cell differentiation.** C2C12 cells were differentiated in DMEM/HG plus 2% HS for 3 days and treated for an additional 24 h with the indicated Pi concentrations. The cells were then collected and processed for the following analyses: (**A**) Number of nuclei per myotube (MYH staining) (**B**) Fusion index (**C**) Myotube length (**D**) Myotube width (**E**) Immunoblot analysis of myogenic differentiation markers (MyoD, myogenin, MYH and troponin I) and cell cycle regulators (p21 and cyclin D1) in whole-cell C2C12 lysates. β-actin was used as loading control. (**F**) RT-PCR analysis of *MyoD1*, *myogenin*, *MYH*, *troponin I*, *p21*, and *cyclin D1*. *GAPDH* was used as loading control. (**G**) Cell cycle phase distributions. Data are presented as means ± SEM. *P < 0.05, **P < 0.01, ***P < 0.001.

To model the clinical conditions observed in cases of acquired hyperphosphatemia, we assessed the effects of high Pi on myogenic differentiation. As shown in Figure 2A–2D, C2C12 cells exposed to 3 or 4 mM Pi for 24 h during the differentiation stage exhibited fewer nuclei per myotube, a lower fusion index (i.e. the number of nuclei in myotubes divided by total number of nuclei in both myotubes and myoblasts), and reduced myotube length and width. In turn, expression of MyoD, myogenin, and MYH was reduced at both the protein and mRNA levels, while decreased expression of Troponin I was detected at the protein but not mRNA level (Figure 2E and 2F). Compared to untreated controls, p21 protein as well as cyclin D1 protein and mRNA were upregulated by high Pi in differentiating C2C12 cells (Figure 2E and 2F). Although most C2C12 cells remained arrested at G1 phase, high Pi treatment (3 or 4 mM) appeared to induce a small increase in the populations at S (p < 0.01) and G2/M (p < 0.05) phase (Figure 2G).

### High Pi decreases MMP and increases oxidative phosphorylation in differentiating C2C12 cells

**Figure 3:**
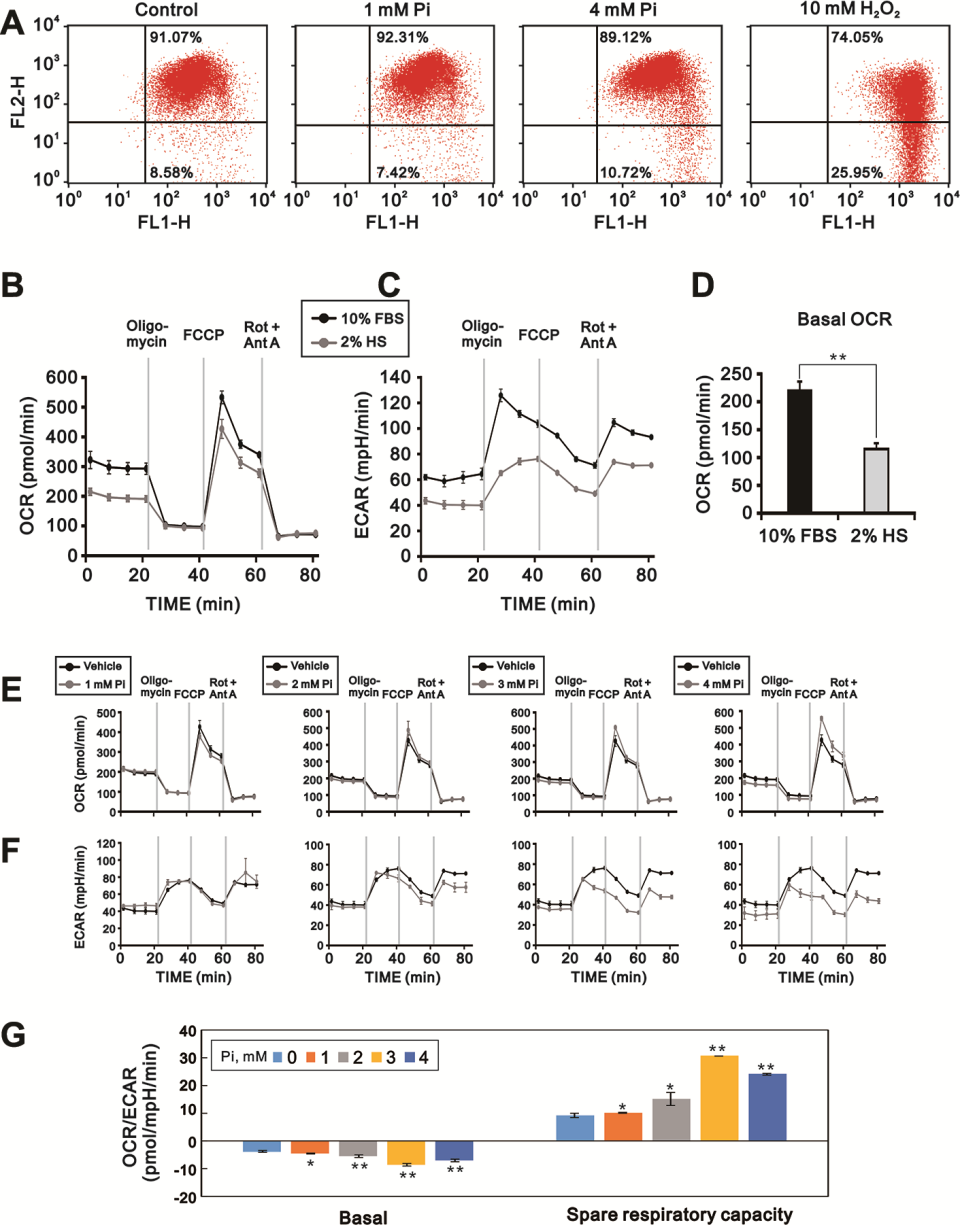
**High Pi impairs mitochondrial function in differentiated C2C12 cells.** Mitochondrial function was assayed by determining MMP, OCR, and ECAR in 3-day-differentiated C2C12 cells treated with the indicated concentrations of Pi for 24 h. (**A**) Flow cytometric analysis of MMP (JC-1 staining). Cells exposed to H_2_O_2_ (10 mM) for 1 h served as positive control. (**B**) OCR (pmol/min) and (**C**) ECAR (mpH/min) were measured in proliferating (10% FBS) and differentiated (2% HS) C2C12 cells using a Seahorse XF24 analyzer. (**D**) Comparison of basal OCR between proliferating and differentiated C2C12 cells. (**E**) OCR and (**F**) ECAR measurements in 3-day-differentiated C2C12 cells treated with the indicated concentrations of Pi for 24 h. (**G**) OCR/ECAR ratios during basal and maximal respiration in differentiated C2C12 cells treated with the indicated concentrations of Pi. Data are presented as means ± SEM. *P < 0.05, **P < 0.01, ***P < 0.001, compared to control (vehicle).

It was previously reported that disruption of the MMP mediates hyperphosphatemia-induced cell dysfunction [14]. To investigate whether mitochondrial dysfunction is involved in high Pi-mediated suppression of C2C12 cell differentiation, we measured MMP, OCR, and ECAR in C2C12 cells exposed to various Pi concentrations. Using flow cytometry and the fluorescent indicator JC-1, we found that MMP was significantly lower in differentiating C2C12 cells exposed to 4 mM Pi than in untreated control cells (p < 0.01; Figure 3A). As shown in Figure 3B–3D, C2C12 cells differentiated under control conditions (2% HS) had a lower basal OCR, an indicator of mitochondrial respiration, and a lower basal ECAR, an indicator of cellular glycolysis, than proliferating cells (10% FBS). In contrast, the OCR/ECAR ratio in differentiated C2C12 cells was significantly increased upon high Pi exposure (p < 0.01), indicating an increase in oxidative phosphorylation (OXPHOS) over glycolysis (Figure 3E and 3F). In addition, both maximum respiration and spare respiratory capacity were increased by high Pi (Figure 3G).

### High Pi induces ROS generation and protein synthesis/degradation imbalance in differentiating C2C12 cells

**Figure 4:**
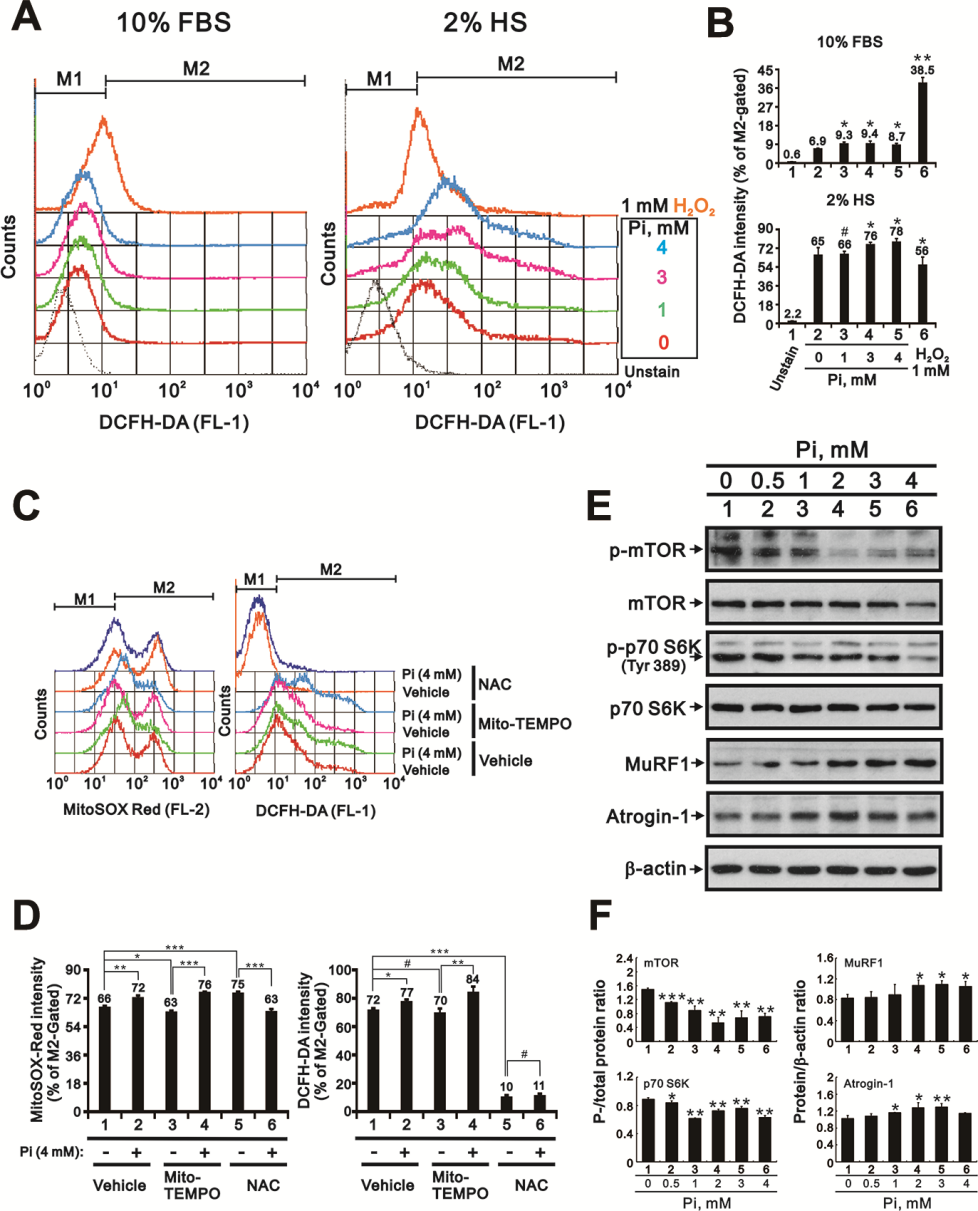
**High Pi induces ROS generation in differentiated C2C12 cells.** (**A**) Analysis of cytosolic ROS levels *via* H2DCFDA flow cytometry in proliferating (10% FBS) and differentiated (2% HS) C2C12 cells treated with the indicated concentrations of Pi for 24 h. (**B**) Bar graph summarizing the data from panel A. Cells exposed to 1 mM H_2_O_2_ served as positive control. *P < 0.05, **P < 0.01 vs. 0 mM Pi. (**C**) Assessment of mitochondrial and cytosolic ROS levels *via* MitoSOX Red and H2DCFDA flow cytometry. Differentiated C2C12 cells were treated for 24 h with 4 mM Pi plus the mitochondria-targeted ROS scavenger Mito-TEMPO (10 μM) or the cytosolic ROS scavenger NAC (10 mM). (**D**) Bar graph summarizing the data from panel (**C**). *P < 0.05, **P < 0.01, ***P < 0.001. (**E**) Representative immunoblot and (**F**) densitometric analyses of protein synthesis (mTOR and S6K) and degradation (MuRF1 and atrogin-1) markers in 3-day-differentiated C2C12 cells treated for 24 h with the indicated concentrations of Pi. *P < 0.05, **P < 0.01, ***P < 0.001 vs. 0 mM Pi. ^#^P > 0.05. Data are presented as means ± SEM.

Because hyperphosphatemia reportedly promotes oxidative stress in cultured insulin-secreting [14] and endothelial cells [16], we wondered whether enhanced ROS production also contributes to high Pi-mediated suppression of skeletal muscle cell differentiation. Measurements of cytosolic ROS levels using the fluorescent indicator H2DCFDA and flow cytometry indicated that basal cytosolic ROS levels were higher in differentiating (2% HS) C2C12 cells than in proliferating (10% FBS) cells (Figure 4A and 4B). This suggested that there is a rise in endogenous ROS levels during *in vitro* differentiation of C2C12 cells. High Pi treatment led to further increases in cytosolic ROS generation, which were greater in differentiating than in proliferating cells (Figure 4A and 4B). Assessment of mitochondrial ROS levels using Mito SOX-Red staining revealed that high Pi (4 mM) also increased mitochondrial ROS generation (Figure 4C and 4D). The increase in cytosolic and mitochondrial ROS levels induced by high Pi could be neutralized with N-acetylcysteine (NAC), a well-known ROS scavenger, but not by Mito-TEMPO, a mitochondrial ROS scavenger (Figure 4C and 4D). Since high ROS levels were shown to induce skeletal muscle wasting by attenuating protein synthesis and accelerating proteolysis [8], we examined by western blotting whether a similar effect occurs in C2C12 cells. Consistent with the referred study, we found that Pi dose-dependently increased protein degradation and decreased protein synthesis, as reflected by lower expression of phosphorylated mTOR and S6K and higher expression of MuRF1 and atrogin-1 (Figure 4E and 4F).

### High Pi induces canonical and non-canonical Nrf2 activation and myogenin suppression in differentiated C2C12 cells

**Figure 5:**
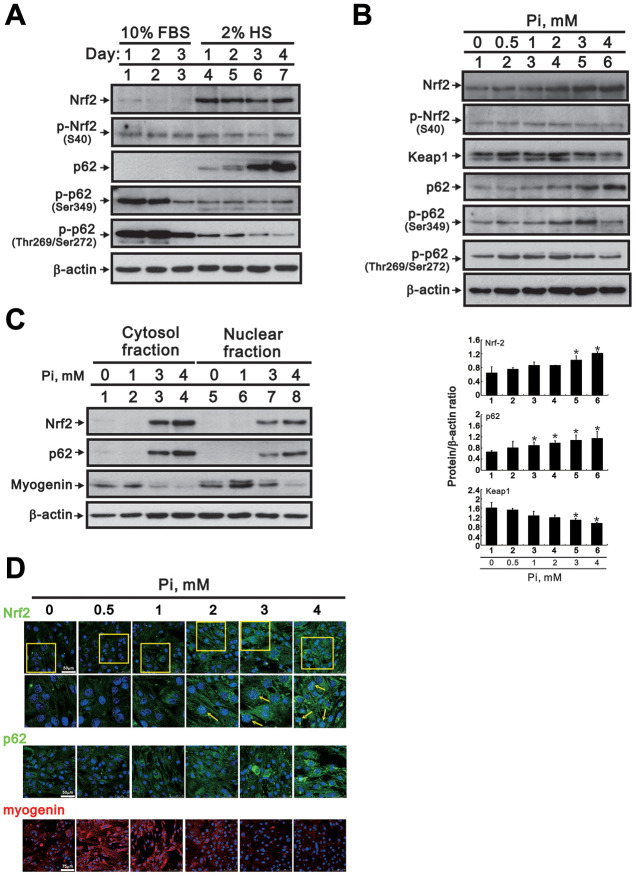
**High Pi activates signaling pathways associated with oxidative stress in differentiated C2C12 cells.** (**A**) Representative Nrf2 and p62 immunoblots from whole-cell lysates prepared from proliferating and differentiated C2C12 cells. β-actin was used as loading control. (**B**) Representative immunoblots (upper panel) and densitometric analysis (lower panel) of Nrf2, Keap1, and p62 expression in whole-cell lysates from 3-day-differentiated C2C12 cells treated for 24 h with the indicated Pi concentrations. Data are presented as means ± SEM. *P < 0.05 vs. 0 mM Pi. (**C**) Representative immunoblots of cytosolic and nuclear Nrf2, p62, and myogenin expression in 3-day-differentiated C2C12 cells treated for 24 h with the indicated Pi concentrations. (**D**) Representative confocal micrographs of Nrf2 (green), p62 (green) and myogenin (red) immunofluorescence in differentiated C2C12 cells treated with the indicated Pi concentrations. Nuclei were stained using DAPI (blue). The boxed areas within Nrf2 staining images are reproduced at higher magnification in the panels immediately below. Arrows highlight positive nuclear Nrf2 expression.

Accumulation of ROS under oxidative stress conditions mediates canonical activation of Nrf2, a central regulator of cellular antioxidant responses, by preventing binding to Keap1, its native repressor [9, 10]. Research has shown that siRNA-mediated inhibition of Nrf2 enhances myogenic differentiation in cultured C2C12 cells, while sulforaphane-induced Nrf2 upregulation has the opposite effect [13]. Using western blotting, we observed that Nrf2 expression increased when C2C12 cells transitioned from the proliferative to the differentiative stage (Figure 5A). Moreover, incubating differentiated C2C12 cells with 0 to 4 mM Pi dose-dependently increased Nrf2 while decreasing Keap1 levels within cells (Figure 5B).

Non-canonical Nrf2 activation can be triggered by Keap1 degradation mediated by the autophagy adaptor p62 [21, 22]. To investigate whether this mechanism is also involved in Nrf2 activation induced by high Pi, we assessed the effect of Pi on p62 expression in differentiating C2C12 cells. We found that p62 expression increased gradually and significantly over the course of differentiation. On the other hand, phosphorylated p62 levels were significantly decreased in differentiating cells (Figure 5A). However, a dose-dependent increase in p62 protein expression occurred upon Pi treatment, with a maximal stimulatory effect on p62 phosphorylation at Ser349 occurring at 3 mM Pi (Figure 5B). Following cellular fractionation, additional immunoblot assays showed that exposure to high Pi led to increased Nrf2 and p62, and reduced myogenin protein levels, both in the nucleus and the cytosol (Figure 5C). These findings were verified by immunofluorescence analyses, which confirmed altered expression of Nrf2, p62, and myogenin in C2C12 cells exposed to high Pi (Figure 5D).

### Blockade of Pi transport, ROS generation, and Nrf2 activation attenuates high Pi-induced Nrf2 activation and myogenin suppression in differentiated C2C12 cells

**Figure 6:**
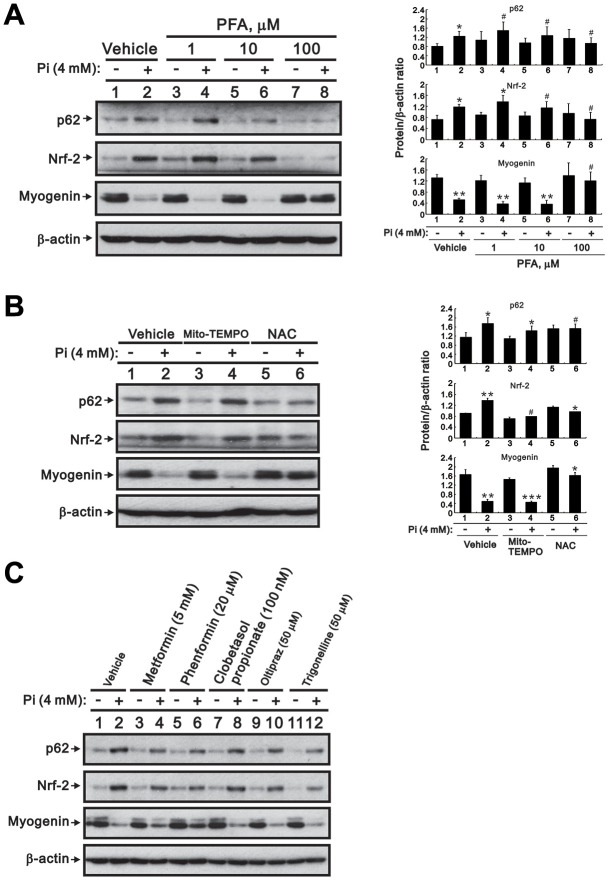
**Inhibition of Pi transport, ROS production, or Nrf2 activity counteracts high Pi-induced changes in Nrf2, p62, and myogenin expression in differentiated C2C12 cells.** (**A**, **B**) Representative immunoblots of p62, Nrf2, and myogenin expression in whole-cell lysates of 3-day-differentiated C2C12 cells treated for 24 h with (**A**) PFA or (**B**) 10 μM Mito-TEMPO or 10 mM NAC. (**C**) Immunoblot analysis of Nrf2, p62, and myogenin expression in whole-cell lysates of 3-day-differentiated C2C12 cells treated for 24 h with 4 mM Pi and various Nrf2 modulators. β-actin was used as loading control. Data are presented as means ± SEM. *P < 0.05, **P < 0.01, ***P < 0.001 vs. corresponding control Pi (-) plus vehicle, Mito-TEMPO, or NAC. ^#^P > 0.05.

To further confirm the impact of high Pi-induced ROS production and Nrf2 activation on skeletal muscle cell differentiation, we treated differentiated C2C12 cells with high Pi combined with a Pi transporter inhibitor, a ROS scavenger, or Nrf2 modulators and determined their effects on the expression of Nrf2, p62, and myogenin. Consistent with our previous observations, the Pi transporter inhibitor phosphonoformic acid (PFA) dose-dependently inhibited the increase in Nrf2 and p62 expression and the reduction in myogenin expression induced by high Pi (Figure 6A). To evaluate whether enhanced ROS generation under high Pi conditions mediates the observed changes in Nrf2, p62, and myogenin expression, we conducted western blot assays after co-application of two antioxidants, Mito-TEMPO and NAC. We found that NAC, but not Mito-TEMPO, attenuated Nrf2 and p62 expression, as well as myogenin suppression, in cells treated with high Pi (Figure 6B). Neither an Nrf2 activator (oltipraz), nor two Nrf2 inhibitors (trigonelline and clobetasol propionate [23]), significantly affected Nrf2, p62, nor myogenin expression in differentiated C2C12 cells treated with or without high Pi (Figure 6C). In contrast, exposure to either metformin or phenformin significantly diminished the stimulatory effect of high Pi on Nrf2 and p62 expression, as well as its inhibitory effect on myogenin expression (Figure 6C).

### Overexpression of Nrf2 increases p62 and decreases myogenin promoter activity

**Figure 7:**
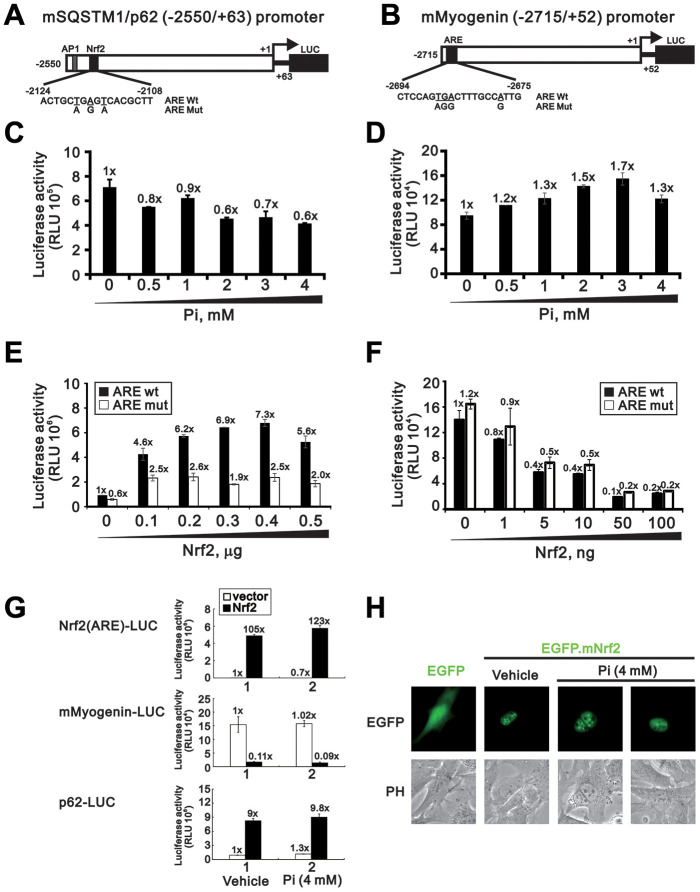
**Nrf2 overexpression increases *p62* and decreases *myogenin* promoter activity.** Schematic diagram of predicted AREs (dark modules) and corresponding mutant ARE sequences within the mouse *p62* (**A**) and *myogenin* (**B**) promoter regions, as determined using Genomatix-MatInspector software. (**C**, **D**) Luciferase reporter assay results. C2C12 cells were transiently transfected with a *mSQSTM1/p62* (-2550/+63)-LUC reporter (**C**) or a *myogenin* (-2715/+52)-LUC reporter (**D**) and then treated for 24 h with the indicated Pi concentrations. (**E**, **F**) Luciferase activity measurements in C2C12 cells transiently co-transfected (24 h) with an Nrf2 expression plasmid plus a *mSQSTM1/p62* (-2550/+63)-LUC (**E**) or a *myogenin* (-2715/+52)-LUC (**F**) reporter plasmid containing wild-type or mutant AREs. (**G**) Luciferase activity measurements in C2C12 cells transiently co-transfected with *Nrf2(ARE)*-LUC, *mSQSTM1/p62* (-2550/+63)-LUC, or *myogenin* (-2715/+52)-LUC reporter plasmids plus an Nrf2 expression plasmid and treated for 24 h with the indicated Pi concentrations. (**H**) Representative fluorescence micrographs of C2C12 cells transfected with 0.5 μg of pEGFP.mNrf2 or pEGFP plasmid DNA in the presence or absence of 4 mM Pi. Data are presented as means ± SEM. PH, phase contrast.

Nrf2 induces the transcription of various antioxidant genes by binding to antioxidant response elements (ARE) in their promoters. The ARE consensus sequence is present in the promoter regions of the mouse *p62* and *myogenin* genes, and previous studies have shown that modulation of Nrf2 expression influences *p62* and *myogenin* promoter activity in C2C12 and HEK 293 cells [13, 24, 25]. To investigate whether Nrf2 transactivates *p62* and *myogenin* gene expression following high Pi treatment, we first made two luciferase reporter constructs driven by ARE sequences in the mouse *p62* and *myogenin* promoters (Figure 7A and 7B). The *p62*-Luc and the *myogenin*-Luc reporter plasmids were alternatively transfected into undifferentiated C2C12 cells, which were then treated for 24 h with 0-4 mM Pi. As shown in Figure 7C and 7D, a decrease in *p62* promoter activity and an increase in *myogenin* promoter activity was observed in a concentration-dependent manner. To further verify that *p62* and *myogenin* are Nrf2 target genes in skeletal muscle cells, we co-transfected a Nrf2 expression vector with *p62*-Luc or *myogenin*-Luc reporter plasmids into undifferentiated C2C12 cells. Results indicated that overexpression of Nrf2 dose-dependently increased *p62* promoter activity while repressing the activity of the *myogenin* promoter (Figure 7E and 7F). Meanwhile, site-directed mutations in the ARE of the *p62* promoter, but not in the ARE of the *myogenin* promoter, significantly diminished Nrf2-induced luciferase expression (Figure 7E and 7F). These data indicate the functionality of the ARE in the *p62*, but not the *myogenin*, promoter. In addition, an assessment of the effects of Nrf2 overexpression and high Pi on the *Nrf2* promoter reporter (*Nrf2(ARE)*-LUC), used as positive control, indicated that the stimulation exerted by Nrf2 on both *p62* and *myogenin* promoter activity may be only marginally affected by Pi levels (Figure 7G). Indicating successful nuclear delivery of Nrf2 following transfection of pEGFP-mouse(m)Nrf2 plasmid, a strong green fluorescent signal, unaffected by high Pi treatment, was detected in cell nuclei (Figure 7H).

### p62-mediated proteasomal degradation and protein aggregation does not participate in high Pi-induced downregulation of myogenin expression

**Figure 8:**
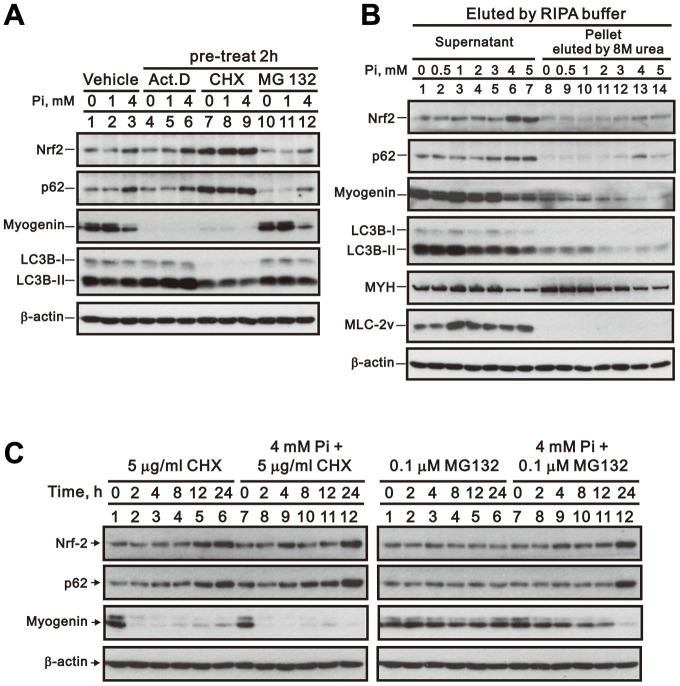
**Inhibition of RNA transcription, protein synthesis, or proteasome formation does not reverse high Pi-induced myogenin downregulation.** (**A**) Representative whole-cell lysate immunoblots for Nrf2, p62, myogenin, and LC3B. Differentiated C2C12 cells were pretreated for 2 h with Act D (10 nM), CHX (5 μg/ml), or MG-132 (0.1 μM) and then exposed for 24 h to the indicated Pi concentrations. (**B**) Immunoblot analysis of Nrf2, p62, myogenin, LC3B, MYH, and MLC-2v content in protein aggregates from differentiated C2C12 cells treated for 24 h with the indicated Pi concentrations. (**C**) Immunoblot analysis of Nrf2, p62 and myogenin expression in whole cell lysates from differentiated C2C12 cells treated for the indicated times with CHX or MG-132, with or without 4 mM Pi.

Actinomycin D (Act D) and cycloheximide (CHX) inhibit de novo RNA and protein synthesis by binding to the transcription initiation complex and by interfering with the translocation step of protein synthesis, respectively [26]. As shown in Figure 8A, treatment of differentiated C2C12 cells with Act D or CHX significantly suppressed myogenin expression independently of Pi, which suggests that in these cells the myogenin mRNA and its encoded protein are both labile. Furthermore, the expression of both Nrf2 and p62 was unaffected and stimulated, respectively, by pre-treatment (2 h) with Act D and CHX (Figure 8A). In turn, pre-treating differentiated C2C12 cells with the proteasome inhibitor MG132 did not alter the inhibitory effect of high Pi on myogenin expression, but slightly reduced Nrf2 and p62 expression (Figure 8A). To further clarify whether the observed reduction in myogenin expression is attributable to increased p62-mediated formation of protein aggregates, we isolated protein aggregates from differentiated C2C12 cells treated with 0 to 5 mM Pi and determined their myogenin content. Western blotting results revealed that Pi treatment induced concentration-dependent increases in Nrf2 and p62 expression, and a decrease in myogenin levels, within protein aggregates from both differentiated C2C12 cell lysates and the corresponding supernatants (Figure 8B). In time-course experiments in differentiated C2C12 cells (Figure 8C), myogenin protein was degraded within 2 h after CHX treatment, while MG132 failed to suppress myogenin degradation induced by high Pi treatment. Meanwhile, the stimulatory effect of high Pi on Nrf2 and p62 protein expression was not affected by MG132 treatment, whereas CHX exposure stabilized Nrf2 and p62 protein levels regardless of the Pi concentration present in the culture medium.

### High Pi diet affects muscle Nrf2, p62, and myogenin expression in both sham and CKD mice

**Figure 9:**
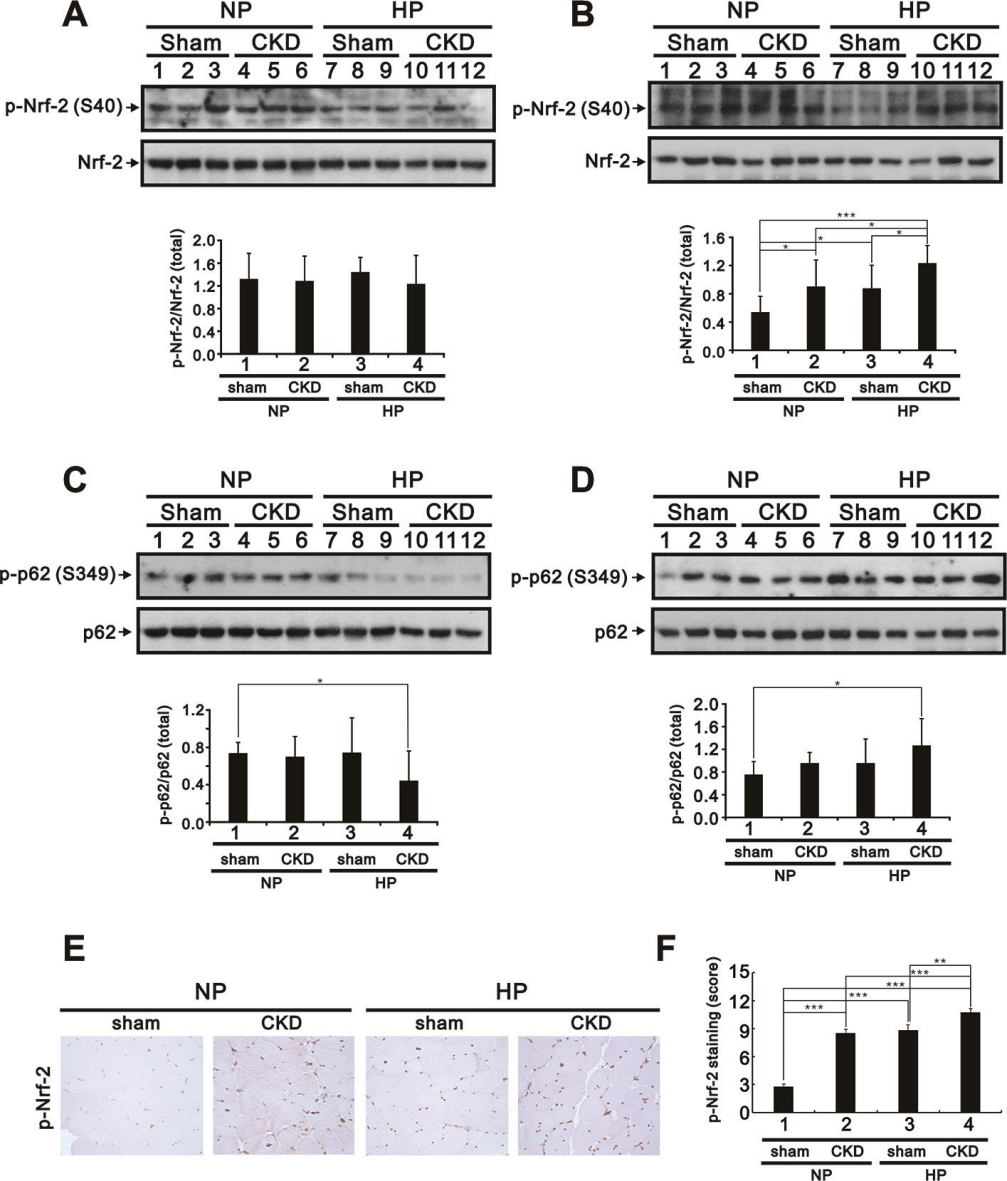
**High-Pi diet alters Nrf2 and p62 expression in GA muscle from sham-operated and CKD mice.** Eight-week-old mice underwent either a sham operation or 5/6 nephrectomy (CKD), after which they were fed a normal Pi (NP) or high-Pi (HP) diet for 20 weeks. Cytosolic (**A** and **C**) and nuclear (**B** and **D**) fractions were extracted from GA muscle samples and probed for Nrf2 and p-Nrf2 (**A** and **B**) and for p62 and p-p62 (**C** and **D**). Levels of these proteins were quantified by computer-assisted densitometric analysis. (**E**) Representative images of p-Nrf2 immunohistochemistry in GA muscle sections. (**F**) Semiquantitative IHC scoring from images like those shown in (**E**). Data are presented as means ± SEM. *P < 0.05, **P < 0.01, ***P < 0.001.

**Figure 10:**
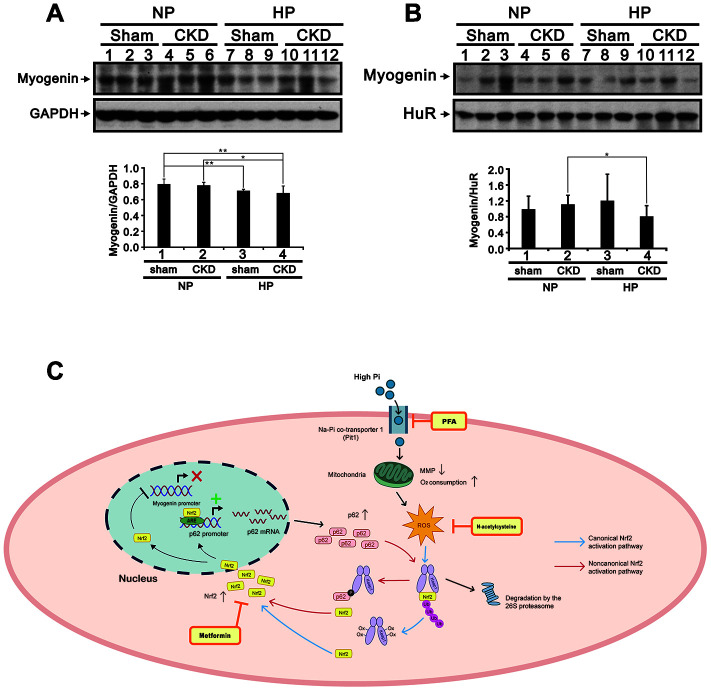
**High-Pi diet alters myogenin expression in GA muscle from sham-operated and CKD mice.** Representative myogenin immunoblots from cytosolic (**A**) and nuclear (**B**) GA muscle fractions. Levels of these proteins were quantified by computer-assisted densitometric analysis. Data are presented as means ± SEM. *P < 0.05, **P < 0.01. (**C**) Schematic illustration of the molecular mechanism by which high Pi represses myogenic differentiation and promotes muscle atrophy. GA, gastrocnemius muscle; PFA, phosphonoformic acid; MMP, mitochondrial membrane potential; ROS, reactive oxygen species; ub-Nrf2, ubiquitinated Nrf2; ARE, antioxidant response element; Ox-Keap1, oxidized Keap1; p-p62, phosphorylated p62.

Muscle atrophy is a common issue in patients with CKD [1]. To investigate whether Pi availability impacts skeletal muscle properties in CKD, we fed sham-operated and 5/6 nephrectomized (CKD) mice a high (2.0%) Pi (HP) diet for 5 months and then assessed blood chemistry, muscle weight, hindlimb grip strength, and Nrf2, p62, and myogenin expression in the gastrocnemius (GA) muscle. Compared to sham-operated mice fed a normal Pi (sham/NP) diet, sham-operated mice fed a HP (sham/HP) diet exhibited significantly higher iPTH and FGF23 levels. In CKD/NP mice, the serum BUN, Cr, Ca^2+^ and iPTH levels were significantly higher than those measured in sham/NP mice. The serum Pi levels in CKD/NP mice (3.01 ± 0.20 mmol/L) were similar to those in sham/HP mice (2.94 ± 0.07 mmol/L) but, although raised in comparison to that of levels in sham/NP mice (2.75 ± 0.10 mmol/L) this difference did not reach statistical significance. CKD/NP mice also exhibited lower body weight (BW), lower GA muscle weight, and a trend toward reduced grip strength compared to sham/NP mice. Feeding CKD mice a HP diet further increased serum Pi, iPTH, and FGF23 levels, but did not affect BW, GA muscle weight, or grip strength (Table 1). As shown in Figure 9A–9D, cytoplasmic levels of phosphorylated Nrf2 (Ser40) in GA muscle samples were similar among groups. On the other hand, nuclear levels of phosphorylated Nrf2 were upregulated in CKD/NP, sham/HP, and CKD/HP mice, with the greatest increase observed in the CKD/HP group. In turn, compared to sham/NP mice, phosphorylated p62 (Ser349) levels were significantly decreased and increased, respectively, in cytosolic and nuclear fractions of GA samples from CKD/HP mice. In line with these immunoblotting results, immunohistochemical assays revealed higher nuclear expression of phosphorylated Nrf2 in GA muscle sections from CKD/NP and sham/HP mice, compared with sham/NP mice. This expression pattern was, in turn, more pronounced in CKD/HP mice (Figure 9E and 9F). Meanwhile, administration of the HP diet decreased cytoplasmic myogenin expression in GA muscles of sham-operated mice, and suppressed both cytoplasmic and nuclear myogenin expression in CKD mice (Figure 10A and 10B).

**Table 1:** Serum biochemistry and gastrocnemius muscle weight and grip strength in WT and CKD mice fed a NP or HP diet.

	**Sham/NP**	**CKD/NP**	**Sham/HP**	**CKD/HP**
**BUN (mg/dL)**	24.16 ± 2.19	64.66 ± 4.97^a,b^	21.00 ± 1.23	38.50 ± 2.29^a,b,c^
**Cr (mg/dL)**	0.21 ± 0.01	0.45 ± 0.04^ab^	0.25 ± 0.02	0.33 ± 0.02^a,b,c^
**Ca^2+^ (mmol/L)**	2.20 ± 0.03	2.48 ± 0.03^a,b^	2.30 ± 0.02^a^	2.27 ± 0.02^c^
**Pi (mmol/L)**	2.75 ± 0.10	3.01 ± 0.20	2.94 ± 0.07	3.48 ± 0.19^a,b,c^
**iPTH (pg/ml)**	328.37 ± 30.61	861.27 ± 35.70^a,b^	1039.49 ± 83.83^a^	2347.08 ± 60.47^a,b,c^
**FGF23 (pg/ml)**	293.05 ± 13.13	559.86 ± 78.07^b^	927.63 ± 200.53^a^	2543.12 ± 111.36^a,b,c^
**BW (g)**	34.15 ± 0.62	28.60 ± 0.42^a,b^	34.50 ± 1.02	26.51 ± 1.18^a,b^
**GA (mg)**	170.11 ± 2.28	163.59 ± 3.97^a^	173.53 ± 2.82	153.90 ± 5.42^a,b^
**GS (N)**	0.75 ± 0.02	0.67 ± 0.03	0.71 ± 0.04	0.61 ± 0.02^a,b^

WT, wild-type; CKD, chronic kidney disease; NP, normal 0.6 % phosphate diet; HP, high 2% phosphate diet; BUN, blood urea nitrogen; Cr, creatinine; Ca^2+^, calcium; Pi, phosphate; iPTH, intact parathyroid hormone; FGF23, fibroblast growth factor 23; BW, body weight; GA, gastrocnemius muscle; GS, grip strength. Data are presented as means ± SEM. N = 6 samples per group.

^a^P < 0.05 versus sham-operated mice on the NP diet; ^b^P < 0.05 versus sham-operated mice on the HP diet; ^c^P < 0.05 versus CKD mice on the NP diet.

## DISCUSSION

Muscle atrophy is both a physiological consequence of aging (i.e. sarcopenia) and a pathological feature of CKD and other diseases. Although a link between hyperphosphatemia and skeletal muscle atrophy has been suggested by some studies, the mechanisms involved are not fully understood. The present study demonstrates that exposing C2C12 skeletal muscle cells to high Pi leads to changes consistent with muscle wasting, namely reduced myotube size, increased ROS generation, decreased protein synthesis, and accelerated protein degradation. High Pi also suppressed normal *in vitro* differentiation of C2C12 cells, evidenced by fewer nuclei per myotube, a lower fusion index, and reduced expression of the differentiation markers myogenin, MyoD, MYH, and troponin I. This suppression was accompanied by an increase in ROS generation and mitochondrial oxygen consumption, upregulation of MuRF1, atrogin-1, Nrf2, and p62 protein expression, and downregulation of mTOR, S6K, and Keap1 protein expression. Pharmacological inhibition of Pi influx, scavenging cytosolic ROS, and inhibition of Nrf2 activity diminished both the inhibitory effect of high Pi on myogenin expression and the stimulatory effect of Nrf2 on p62 expression. Suggesting a key regulatory role of Nrf2 in the suppression of C2C12 cell differentiation observed under high Pi conditions, the activity of the *myogenin* and *p62* gene promoters was decreased and increased, respectively, by Nrf2 overexpression. In turn, administration of a high Pi diet led to higher serum iPTH and FGF23 levels and increased Nrf2/p62 expression and reduced myogenin expression in GA muscles, without affecting body weight, GA muscle weight, or grip strength in both sham-operated and CKD mice. These data suggest that high Pi suppresses skeletal muscle cell differentiation and induces muscle atrophy through induction of oxidative stress and activation of Nrf2/p62 signaling. A schematic summary of our findings is shown in Figure 10C.

Maintaining adequate serum Pi levels is essential for normal skeletal muscle function. A recent study using *in vivo* saturation transfer ^31^P-magnetic resonance spectroscopy demonstrated that rates of muscle ATP synthetic flux (*V*_ATP_) were decreased in mice with diet-induced or genetically induced (*NaPi2a^−/−^*) hypophosphatemia and were restored after normalization of serum Pi levels through oral Pi repletion [27]. Similar findings were obtained in a patient carrying a *SLC34A3* mutation who presented with chronic hypophosphatemia [27]. On the other hand, several *in vivo* and *in vitro* studies have also suggested a correlation between hyperphosphatemia and skeletal muscle atrophy. For example, Ohnishi et al. recently reported that genetic ablation of the Pi-regulating hormones Klotho and FGF23 in mice leads to hyperphosphatemia and muscle atrophy that can be reversed by a low-Pi diet or genetic ablation of *SLC34A1* [6]. In addition, exposing rat L6 myotubes to high- Pi medium reportedly leads to a reduction in cell diameter [28]. Consistent with those earlier observations, we found that exposure of differentiated C2C12 cells to a high-Pi medium decreases myotube size, the number of nuclei per myotube, and the fusion index. High Pi treatment also reduced MyoD, myogenin, MYH, and troponin I expression, and caused a reduction in myogenin levels that was reversed by Pi transporter blockade using PFA. Further supporting the idea that high Pi suppresses myogenic differentiation and induces muscle wasting, a high Pi diet induced a significant reduction of myogenin expression in cytoplasmic fractions of sham-operated GA muscles and in both nuclear and cytoplasmic fractions of GA muscle samples from CKD mice.

Excess ROS production has been shown to contribute to the pathogenesis of skeletal muscle wasting by disturbing the balance between protein synthesis and degradation [8]. In the present study, we observed that incubating differentiated C2C12 cells in a high Pi medium increased cytosolic and mitochondrial ROS levels. ROS elevation was accompanied by decreases in mTOR and S6K1 and increases in atrogin1 and MuRF1 expression, which suggests that ROS-mediated decreases in protein synthesis and increased proteolysis may contribute to muscle atrophy associated with high Pi. Both the increase in intracellular ROS generation, as well as reduction in myogenin expression observed in high Pi-treated, differentiated C2C12 cells were reversed by the ROS scavenger NAC, which was previously shown to reduce muscle fatigue and improve muscle performance during exercise programs [29–31]. Furthermore, as in insulin-secreting cells [14], high Pi disrupted MMP and increased OXPHOS in differentiated C2C12 cells, indicating that high Pi-mediated mitochondrial dysfunction may be an important source of ROS generation.

Under oxidative stress conditions Keap1-mediated Nrf2 ubiquitination is impeded, allowing Nrf2 to translocate into the nucleus and to initiate the transcription of genes containing a cis-acting ARE in their promoters [10]. Our studies indicate that high Pi, likely acting through oxidative stress, induces canonical activation of the Nrf2 pathway in C2C12 skeletal muscle cells *in vitro*. Overexpression of Nrf2 suppressed *myogenin* promoter activity, while treatment with the Nrf2 inhibitors metformin and phenformin attenuated the suppressive effect of high Pi on myogenin protein expression. In line with these observations, increases in Nrf2 activity induced by methysticin or sulforaphane reportedly suppress *myogenin* promoter activity in satellite cells and myogenin expression in C2C12 cells [13, 24]. Conversely, inhibition of Nrf2 using siRNA stimulates myogenic differentiation in C2C12 cells [13]. Modulation of Nrf2 activity also influences muscle regeneration in a murine model of hind limb ischemia–reperfusion injury [24] and disrupts the contractile and metabolic properties of skeletal muscle in streptozotocin-induced diabetic mice [13]. In addition, recent studies have shown that Nrf2 may be activated through a non-canonical, p62-mediated pathway [10, 22, 32]. In that regard, we observed that high Pi stimulated p62 expression, Nrf2 expression and nuclear translocation, and decreased Keap1 levels in differentiated C2C12 cells. This suggests that p62 upregulation may contribute to non-canonical activation of Nrf2 under high Pi conditions. Moreover, our finding that *p62* promoter activity was stimulated by overexpression of Nrf2, while mutation of the corresponding ARE sequence abolished promoter activation suggests that high Pi induces a positive feedback loop between p62 and Nrf2 in C2C12 cells.

p62 modulates protein expression by targeting ubiquitinated proteins to the 26S proteasome and by shuttling misfolded proteins to the aggresome [17, 32, 33]. Since the proteasome inhibitor MG132 did not prevent the high Pi-mediated decrease in myogenin expression, and the insoluble protein pellets extracted from high Pi-treated C2C12 cells were not enriched in myogenin, it appears that neither p62-mediated proteasomal degradation nor aggresome formation is involved in the downregulation of myogenin expression induced by high Pi. On the other hand, CHX increased Nrf2 and p62 protein stability and further suppressed myogenin expression in high Pi-treated, differentiated C2C12 cells. These findings suggest that protein degradation modulates the stimulatory effect of high Pi on Nrf2 and p62 protein expression in skeletal muscle cells.

Muscle wasting is a major clinical issue in CKD patients, where preservation of adequate muscle mass and function are critical concerns [1, 34]. The accumulation of uremic toxins caused by decreased renal excretion is responsible for the dysfunction of multiple organs during the course of progressive CKD [35]. Recent studies demonstrated that indoxyl sulfate, a protein-bound uremic toxin, negatively affects skeletal muscle cells by enhancing ROS production [36, 37] and inducing mitochondrial dysfunction [38]. Emerging evidence suggests that excess Pi should also be considered an uremic toxin, as it can induce various adverse effects, such as vascular calcification, endothelial dysfunction, and impaired bone mineralization, that accelerate aging and contribute to the progression of CKD [15, 39, 40]. Although we demonstrated that high Pi exerts a detrimental effect on cultured skeletal muscle cells, our *in vivo* findings did not completely recapitulate what we observed *in vitro*. Feeding 10-week-old sham-operated and CKD mice with an HP diet for 20 weeks had no obvious effects on BW, GA muscle weight, and grip strength, but significantly increased serum iPTH, a known muscle-wasting factor [41] compared to their NP diet-fed counterparts. Furthermore, feeding the HP diet increased nuclear levels of phosphorylated Nrf2 in GA muscles from sham-operated and CKD mice, and increased nuclear levels of phosphorylated p62, while decreasing nuclear myogenin levels, in GA muscles from CKD mice. These findings support the notion that systemic hyperphosphatemia triggers pathological signaling *via* the p62/Nrf2 axis, which may contribute to the pathogenesis of muscle atrophy during CKD progression. Notably, a recent animal study showed that feeding 20- to 24-week-old WT mice a HP diet for 12 weeks induced exercise intolerance and altered fatty acid metabolism in skeletal muscles [42]. Thus, we speculate that more definite symptoms of hyperphosphatemia-induced muscle wasting will be observed in older HP diet-fed mice. Indeed, a cross-sectional study analyzing data from the US National Health and Nutrition Examination Survey reported that higher Pi quartiles correlated with lower muscle strength in people older than 65 years and predicted a greater risk for dynapenia [43]. These data suggest that age might be a crucial factor modulating the effects of hyperphosphatemia on skeletal muscle function. Further studies are required to determine whether counterbalancing mechanisms are activated to mitigate the detrimental effects of high Pi on skeletal muscle function.

In summary, our study suggests that high Pi suppresses myogenic differentiation *in vitro* and promotes skeletal muscle atrophy *in vivo*, especially in diseases associated with muscle wasting such as CKD. These effects entail enhanced Nrf2 transcriptional activity through both canonical (*via* ROS) and non-canonical (*via* p62) pathways. Keeping serum Pi levels within a normal range may help prevent skeletal muscle dysfunction both during aging and in muscle-wasting conditions, while inhibition of Pi entry into cells, cytosolic ROS production, or Nrf2 activation may ameliorate the negative effects of high Pi on skeletal muscle cells.

## MATERIALS AND METHODS

### C2C12 myotube culture and Pi treatments

Mouse C2C12 myoblasts seeded into 6-well plates at a density of 2x10^5^ cells/well were first cultured for 3 days in DMEM/HG [Dulbecco's modified Eagle medium/high glucose (4500 mg/L)] supplemented with 10% fetal bovine serum (FBS, Gibco) at 37°C and 5% CO_2_. They were then cultured for an additional 4 days in differentiation medium consisting of DMEM/HG supplemented with 2% adult horse serum (HS, Gibco). During this period the spindle-shaped C2C12 myoblast cells differentiated and fused, gradually forming multinucleate myotubes. The myotubes thus formed were then incubated for 24 h in sodium phosphate buffer (1M Na_2_HPO_4_/NaH_2_PO_4_, pH 7.4) titrated to achieve final Pi concentrations of 0, 0.5, 1, 2, 3, or 4 mM. Thereafter, the myotubes were harvested or lysed for downstream analyses. A minimum of three independent experiments were performed for each condition.

### Cell cycle, MMP, and ROS generation assays

Cell cycle phase distributions were determined by measuring cellular DNA content using fluorescence-activated cell sorting (FACS). Cells were harvested and fixed in 70% ice-cold ethanol and kept at 20°C overnight. Before analysis, the cells were washed twice with ice-cold PBS and stained with propidium iodide (PI; 5 μg/ml in PBS, 1.80.5% Triton X-100, and 0.5 μg/ml RNase A) for 30 min at room temperature in the dark. Samples were analyzed using a FACS Calibur flow cytometer (BD Biosciences, CA, USA). Data were analyzed using Cell Quest Pro software (BD Biosciences).

MMP was measured using a BD™ MitoScreen Flow Cytometry Mitochondrial Membrane Potential Detection Kit (BD Biosciences) according to the manufacturer’s instructions. In brief, C2C12 cells were seeded onto 6-well plates and cultured to a density of 1.8x10^5^ cells/ml. After exposure to different Pi concentrations for 24 h, the cells were stained with JC-1 staining reagent (5,5’,6,6’-tetrachloro-1,1’,3,3’-tetraethylbenzimi-dazolyl carbo-cyanine iodide) for 15 min at 37°C in a CO_2_ incubator. The cells were then washed, and JC-1 fluorescence was measured using a flow cytometer. The excitation wavelength was 488 nm, and the emission wavelengths were 530 nm (FL1-H channel) for monomers and 580 nm (FL2-H channel) for aggregates. Cytosolic and mitochondrial ROS levels in Pi-treated C2C12 myotubes were measured using the fluorescent indicator H2DCFDA (2’,7’-dichlorodihydrofluorescein diacetate) and MitoSOX™ Red assay, respectively. Myotubes treated for 24 h with the indicated concentrations of Pi were stained with 10 μM H2DCFDA or 5 μM MitoSOX™ Red at 37°C. After incubation for 30 min, ROS levels were assessed by flow cytometry at excitation/ emission wavelengths of 488/525 nm.

### Measurement of oxygen consumption rate (OCR) and extracellular acidification rate (ECAR)

Cellular OCR and ECAR were measured using XF24 bioenergetic assays according to the manufacturer’s instructions (Seahorse Biosciences, Billerica, MA, USA). Briefly, C2C12 cells were suspended in DMEM containing 10% FBS and then seeded onto an XF24 microplate. For C2C12 myotube differentiation, growth medium was changed to DMEM/HG with 2% HS, and the cells were incubated for an additional 3 days, after which the assays were initiated by removing the exhausted medium and replacing it with sodium bicarbonate-free DMEM (pH 7.4) containing 2% FBS and 2% HS. OCR and ECAR were detected at steady state. Oligomycin (1 μM), carbonyl cyanide 4-[trifluoromethoxy] phenylhydrazone (FCCP; 0.5 μM), and rotenone/antimycin A (Rot-Ant A; 0.5 μM) were added sequentially to the wells to determine the maximal and non-mitochondrial respiration rates.

### Whole-cell, cytoplasmic, and nuclear extract preparations

C2C12 cells (2x10^5^ cells/well) were grown to confluence in 100 mm culture dishes and collected by Trypsin–EDTA. To prepare whole-cell extracts, C2C12 cells and gastrocnemius (GA) muscle samples were lysed in lysis buffer [50 mM HEPES (pH 7.6), 150 mM NaCl, 0.5% Triton X-100, 10% glycerol, and 0.1% dodecyl sulfate] at 4°C and centrifuged for 15 min at 14,000×g, 4°C. Cytoplasmic and nuclear extracts from C2C12 cells were prepared with a ProteoExtract® Subcellular Proteome Extraction Kit (Merck Millipore, MA, USA). Cytoplasmic and nuclear extracts from GA samples were prepared with a Subcellular Protein Fractionation Kit for Tissues (Thermo Fisher Scientific, CA, USA).

### Western blotting

C2C12 cells and GA samples were lysed in lysis buffer [50 mM HEPES (pH 7.6), 150 mM NaCl, 0.5% Triton X-100, 10% glycerol, and 0.1% dodecyl sulfate] for 30 min at 4°C. The resultant protein extracts were separated by SDS-PAGE and electrotransferred onto PVDF membranes (Immobilon-P; Millipore, MA, USA). The membranes were then incubated with primary antibodies against proteins of interest (Supplementary Table 1). Following addition of HRP-conjugated secondary antibodies, immunoreactive bands were detected using enhanced chemiluminescence (Pierce, Rockford, IL, USA). To analyze protein expression in cellular protein aggregates, total protein was extracted into RIPA buffer, centrifuged, and the pellet collected and eluted with 8 M urea. To normalize protein expression, beta-actin (β-actin), glyceraldehyde-3-phosphate dehydrogenase (GAPDH), and HuR were used as an internal controls for whole-cell lysates, cytoplasmic extracts, and nuclear extracts, respectively. Pixel density scanning using Molecular Analyst software (Bio-Rad, Hercules, CA, USA) was applied for quantification of immunopositive bands.

### Reverse transcription-polymerase chain reaction (RT-PCR)

Total RNA was isolated using TRIsure (Bioline, London, UK) reagent according to the manufacturer’s instructions. For 1^st^-strand cDNA synthesis, 1 μg of total RNA was reverse transcribed using 200 U of M-MLV reverse transcriptase (Epicentre Biotechnologies, WI, USA) for 60 min at 37°C. Specific primers (Supplementary Table 2), dNTPs, and Taq DNA polymerase were added for subsequent PCR reactions on a Veriti Thermal Cycler (Applied Biosystems, MA, USA). GAPDH was used as an internal control to normalize gene expression.

### Immunohistochemistry

GA muscles were collected and fixed in 4% paraformaldehyde for 24 h. The samples were then embedded in paraffin and transversely sectioned (4 μm) from the mid-belly of the muscles. For immunohistochemical staining, deparaffinized tissue sections were incubated with primary antibody against phosphorylated Nrf2 (Abcam, Cambridge, MA, USA) at 4°C overnight. The sections were then washed in PBST and incubated with HRP Labeled Polymer (Dako, Glostrup, Denmark) for 1 h. Antigen-antibody complexes were visualized using DAB chromogen (Dako) followed by hematoxylin counterstain. Semiquantitative analysis of tissue immunoreactivity was done by two pathologists using an immunohistochemical rating score (IRS). Five randomly selected fields per tissue section per mouse were evaluated using high power (×400) microscopy. In brief, the intensity was rated as 0, no staining; 1, weak staining; 2, moderate staining; and 3, strong staining. The quantity of positively stained cells for each section was defined as 0, no cells; 1, <10% 2, 10% to 50% 3, 51% 80% and 4, <80%. The IRS was obtained by multiplying the percentage of positive cells by the staining intensity.

### Immunofluorescence

Cells were grown on coverslips and fixed for 6 min in 4% paraformaldehyde at room temperature. After washing with PBS, the cells were permeabilized for 6 min using 0.1% Triton X-100, blocked in 1% BSA for 1 h, and incubated for 1 h at room temperature with primary monoclonal antibodies: anti-Nrf2, sc-365949; anti-SQSTM1/p62, sc-28359 (Santa Cruz Biotechnology, Inc., Dallas, TX, USA), and anti-myogenin, ab124800 (Abcam, Cambridge, MA, USA) diluted in blocking buffer. After washing the cells 3 times with PBS and 2 times with blocking buffer, fluorescently-labeled secondary antibodies diluted in blocking buffer were applied for 30-45 min at room temperature in the dark. The cells were then washed with PBS, and the coverslips mounted on glass slides for microscopy.

### Plasmid DNA construction and luciferase reporter assays

Full-length mouse *Nrf2* c-DNA was inserted into pSG5.HA and pEGFP vectors *via* the *Xho*I-*Kpn*I restriction site. Mouse wild-type (WT) *SQSTM1/p62 and myogenin* promoter fragments were inserted into a pGL3-LUC vector at the *BamH*I/*Xho*I site. The Nrf2(ARE)-LUC reporter was purchased from Signosis Inc. (Cat# LR-2106, Santa Clara, CA, USA). In turn, two reporter constructs containing regions located -2550 to +63 base pairs (bp) in the mouse *p62* promoter and -2715 to +52 bp in the mouse *myogenin* promoter were generated. Mutant ARE in the *SQSTM1/p62* and *myogenin* promoters were constructed through PCR-based site-directed mutagenesis (Figure 7A and 7B, respectively).

Luciferase activity was monitored in C2C12 cells transfected over 24 h with the reporter plasmids (0.25 μg) using a Dual-Luciferase Reporter Assay Kit (Promega, MI, USA) according to the manufacturer’s instructions [44]. Luciferase activities in cell extracts are presented as relative light units (RLU) and expressed as the mean ± standard deviation of results from three independent cell cultures.

### Animal experiments

Eight-week-old male C57B6 mice (25-30 g) were housed in a light- and temperature-controlled room with *ad libitum* access to deionized drinking water and standard chow. After a 1-week acclimatization period, the animals were randomly assigned to one of two groups (n = 12 mice/group) and subjected to either a sham operation or two-stage 5/6 nephrectomy (5/6 Nx; to model CKD) under ketamine/xylazine anesthesia (90/9 mg/kg, *i.p.*). During the first stage of nephrectomy, approximately two thirds of the left kidney were removed after ligation of the upper and lower poles. One week later, the entire right kidney was removed after ligation of the vascular pedicle. The sham operation involved a flank incision and gentle manipulation of the kidney, without altering its structure, on the same day that mice in the CKD group were subjected to the first stage of nephrectomy. Both sham-operated and 5/6 Nx mice were then assigned to two subgroups (n = 6 mice/group) and fed, respectively, a high-Pi (2% Pi) and a normal-Pi (0.6% Pi) diet. After 5 months on the respective diets, mouse hind limb grip strengths were measured using a MK-380 grip strength meter (Muromachi Kikai Co., Ltd., Tokyo, Japan). Serum samples were collected for analysis of BUN, Cr, Ca^2+^, Pi, iPTH, and FGF23. GA muscles from the sacrificed mice were weighed and processed for immunoblot analysis of Nrf2, p62, and myogenin expression. The Animal Ethics Board of the National Defense Medical Center (Taipei, Taiwan) approved all animal experimental procedures.

### Statistical analyses

All values are given as means ± SEM unless stated otherwise. Unpaired Student’s t-tests were used for two-group comparisons, and one-way analysis of variance was used for comparisons among multiple groups. All analyses were performed using GraphPad Prism version 5 software (GraphPad Software Inc., CA, USA). P < 0.05 was considered significant.

## Supplementary Material

Supplementary Tables
