# Supplementary material for: High phosphate induces skeletal muscle atrophy and suppresses myogenic differentiation by increasing oxidative stress and activating Nrf2 signaling
Source: Aging (Albany NY). 2020 Nov 2;12(21):21446–68. doi: 10.18632/aging.103896 (PMC7695395; doi:10.18632/aging.103896)
Supplement: Supplementary Tables [file aging-12-103896-s001..pdf]

SUPPLEMENTARY TABLES

Supplementary Table 1. List of the antibodies used for immunoblot analyses.

**Santa Cruz:**		
MYH	sc-376157	1:2000
Cyclin D1	sc-718	1:1000
p21	sc-397	1:1000
beta-actin	sc-47778	1:10000
Myo D	sc-304	1:1000
Nrf-2	sc-365949	1:1000
p62	sc-28359	1:1000
**Abcam:**		
Myogenin	ab124800	1:2000
p-Nrf-2 (S40)	ab76026	1:1000
MuRF	ab77577	1:1000
Atrogin-1	ab74023	1:1000
**Cell signaling:**		
Troponin I	#4002	1:1000
p-p62 (S349)	#95697	1:1000
p-p62 (Thr269/Ser272)	#13121	1:1000
Atg7	#8558	1:1000
Beclin-1	#3495	1:1000
LC3B	#2775	1:1000
p-p70 S6K (Tyr389)	#9205	1:1000
p70 S6K	#2708	1:1000
p-mTOR	#2971	1:1000
mTOR	#2972	1:1000
MLC2v	#8505	1:1000
**Proteintech:**		
Keap1	60027-I-Ig	1:1000

Supplementary Table 2. PCR primers used in this study.

Gene name	Primer (5'→3')
myogenin	Forward: 5'-CTACCTTCCTGTCCACCTTC-3'
	Reverse: 5'-CTCCAGTGCATTGCCCCACT-3'
MYH	Forward: 5'-GCCTCATCCACACCAAGAAGA-3'
	Reverse: 5'-TCCACCAGATCCTGCAATCTC-3'
Troponin I	Forward: 5'-GCAAAAGTCACCAAGAACATC-3'
	Reverse: 5'-GCGCCAGTCTCCCACCTCCCGG-3'
p21	Forward: 5'-GCTGTCTCCAGGAGGCCCG-3'
	Reverse: 5'-GCTGGTCTGCCTCCGTTTTCG-3'
cyclin D1	Forward: 5'-ATGGAACACCAGCTCCTGTG-3'
	Reverse: 5'-CTTAGAGGCCACGAACATGC-3'
Myo D1	Forward: 5'-CAGCGGGCACCACCAG-3'
	Reverse: 5'- ATGCTGGACAGGCAGTC-3'
GAPDH	Forward: 5'-CTTCATTGACCTCAACTAC-3'
	Reverse: 5'-GCCATCCACAGTCTTCTG-3'
